# Migration of copper(II) ions in humic systems—effect of incorporated calcium(II), magnesium(II), and iron(III) ions

**DOI:** 10.1007/s11356-024-34758-w

**Published:** 2024-08-21

**Authors:** Martina Klučáková, Vojtěch Enev

**Affiliations:** Brno University of Technology, Faculty of Chemistry, Purkyňova 118, 612 00 Brno, Czech Republic

**Keywords:** Humic acid, Hydrogel, Copper, Calcium, Magnesium, Iron, Diffusion, Immobilization

## Abstract

The mobility of heavy metals in natural soil systems can be affected by the properties and compositions of those systems: the content and quality of organic matter as well as the character of inorganic constituents. In this work, the diffusion of copper(II) ions in humic hydrogels with incorporated calcium(II), magnesium(II), and iron(III) ions was investigated. The methods of instantaneous planar source and of constant source were used. Experimental data yielded the time development of the concentration in hydrogels and the values of effective diffusion coefficients. The coefficients include both the influence of the hydrogel structure and the interaction of diffusing particles with the hydrogel. Our results showed that the presence of natural metal ions such as calcium, magnesium, or iron can strongly affect the diffusivity of copper in humic systems. They indicate that the mobility of copper ions depends on their concentration. The mobility can be supported by higher contents of copper in the system. While the incorporation of Ca and Mg resulted in the decrease in the diffusivity of copper ions, the incorporation of Fe(III) into humic hydrogel resulted in an increase in the diffusivity of Cu(II) in the hydrogel in comparison with pure humic hydrogel.

## Introduction

Humic substances as the major components of soil organic matter play an important role in the bioavailability, toxicity, and transport of different organic and inorganic pollutants in nature (Ding et al. 2019; Hayes et al. 2008, 2012; Sureshkumar et al. 2013). Transport processes are therefore important for understanding the complex behaviour of natural systems and the role of their constituents. The environmental behaviour of metal ions is strongly affected by their binding to humic substances (Klučáková and Pekař 2003b, 2004; Paradelo et al. 2012; Saito et al. 2005).

Humic substances as major constituents of soil organic matter form complexes with metal ions and tend to control their speciation, toxicity, and bioavailability (Gao et al. 2015; Kogut and Voelker 2001; Tipping et al. 2008). Because of the high affinity of metal ions to humic substances, the formed complexes are stable. Martyniuk and Wieckowska (2003) determined the highest affinity to humic acids for Pb, Ag, Hg, Cu, Ba, and Cd. Ding et al. (2019) investigated the following order: Pb > Cu > Ni > Cd > Zn. In contrast, Dinu (2015) demonstrated that Fe and Al ions have higher conditional stability constants than Pb, Cu, and Zn.. van Oort et al. (2020) monitored Zn and Pb contents in 173 soil samples and considered Pb as a less-mobile metal in comparison with Zn. Metal ions may distribute among more types of binding sites. A few of them can be kinetically labile under specific experimental conditions, such as weak bidentate sites for Cu (Shi et al. 2016). The transport of metal–humic complexes can be enhanced when the complexes are mobile in the soil solution or retarded when the organic matter is attached to a solid matrix (Bryan et al. 2005; Paradelo et al. 2012).

The behavior of metal ions and their complexes with humic substances in soils are strongly affected by other different factors such as the pH and ion composition of the solution in pores. The mobility of metal ions as well as their immobilization by complexation with humic substances should thus be influenced by the presence of other ions and substances. Knowledge of the migration behavior of metal ions and their humic complexes under different circumstances is crucial for an understanding of this complex problem.

Copper is an essential nutrient element for plant growth, but its excessive amounts can result in its accumulation in soil and cause risks to the ecosystem as well as to human health (Christl et al. 2005; Wu and Hendershot 2010). Wu and Hendershot (2010) investigated the accumulation of copper in soil, and its toxicity. They showed that increased contents of calcium can suppress the uptake of copper. The effect of calcium and other ions on the complexation of copper in soil and copper toxicity was described in several studies. Kogut and Voelker (2001) studied the copper binding behavior of terrestrial humic substances in seawater. They found that copper binding was weaker than the binding reported for freshwater, possibly indicating the effects of calcium and magnesium, or (in general) ionic strength.

In contrast, Hamilton-Taylor et al. (2002) reported that the presence of seawater concentrations of calcium and magnesium had no detectable effect on the binding of copper, although its binding decreased generally with increasing ionic strength. Their results indicated that the competition for humic binding sites between calcium and magnesium may be important for weakly-binding metals but not for strongly-binding metals such as copper. Kang et al. (2010) studied the adsorption of copper on humic-like substances extracted from activated sludge. Their results confirmed competitive adsorption between free copper and calcium at lower calcium concentrations. Higher calcium concentrations resulted in its precipitation. Gao et al. (2015) observed a decrease in copper binding on dissolved organic matter in the presence of calcium as a result of the decrease in available binding sites.

Saito et al. (2005) analyzed copper binding in a system containing humic acids and goethite. Binding was enhanced in the system containing goethite. Ding et al. (2019) fractionated humic acids by ferrihydrite and studied the binding characteristics of heavy metals. Their results showed that changes in the reactivity of humic acids during their fractionation on ferrihydrite affected the kinetic behavior of heavy metals in solution. Similarly, Lippold et al. (2007) concluded that the competitive influence of iron on the transport of metal ions in water is kinetically controlled. Farina et al. (2018) precipitated ferrihydrite and goethite organo-mineral complexes with humic acids as an analogue of natural organic matter and measured the adsorption of copper. Their results showed that copper adsorption was additive if the mineral fraction predominated in the prepared complexes, and non-additive if the organic fraction predominated. Muller and Cuscov (2017) studied the copper-binding capacity of iron-rich humic substances during transport in marine. The interactions resulted in the decrease in the copper-binding capacity of humic substances. Christl et al. (2001, 2005) demonstrated that adsorption isotherms of copper are independent of concentrations of humic and fulvic acids. In contrast, the binding capacity was affected by pH and ionic strength.

As can be seen, many authors deal with the effects of some ions such as iron, calcium, magnesium, and sodium on the binding of copper or other heavy metals in systems containing humic substances as representatives of natural organic matter. Many authors are interested in adsorption experiments. Studies of interactions of metal ions with humic substances or organic matter directly during their diffusion (or, generally, transport) are relatively scarce. Paradelo et al. (2012) studied the transport of humic substances and copper ions in pore water by means of experiments in water saturated columns filled by quartz sand. They showed that the transport of copper was coupled with humic substances at neutral pH and occurred mostly as free copper ions at pH = 5. Some diffusional characteristics of metal ions in several types of hydrogels were published in connection with the monitoring of metal ions in nature by means of the DGT technique (Scally et al. 2006; Zhang and Davison 1999).

Our approach to the mobility of metal ions is different. We are interested in the diffusion of metal ions through a gelled humic medium, which can be considered as a model of natural soil systems (Klučáková and Pekař 2003b, 2004, 2009). This means that metal ions diffuse through the hydrogel and the front of diffusing ions penetrates into the hydrogel containing non-occupied binding sites. Metal ions can thus interact immediately with humic acids and be (partially) immobilized. These interactions between metal ions and humic binding sites can achieve an equilibrium between immobilized and free mobile ions able to continue in motion.

Our previous work has shown that the binding capacities of humic substances are affected by their used form (Klučáková 2014; Klučáková and Pekař 2003a) and content of active sites (Klučáková 2014; Klučáková and Kalina 2015). Humic substances can be prepared in hydrogel form in several ways. One possibility is the precipitation of humic substances dissolved in alkaline medium by an acid solution, as used in our previous research (Klučáková 2014; Klučáková et al. 2014; Klučáková and Kalina 2015; Klučáková and Pekař 2003b, 2004, 2009). The cross-linking of hydrogels can also be achieved by means of multivalent ions (Klučáková and Pekař 2009). This approach can be used for the preparation of hydrogels containing (besides humic substances) ions characteristic of natural systems (e.g., Fe, Mg, Ca) and better simulate real circumstances in soils.

The main goal of this study was to investigate the diffusion of copper(II) ions in humic hydrogels enriched by calcium(II), magnesium(II), and iron(II) ions; determine the diffusion coefficients; and consider the effect of added ions in comparison with “pure” humic hydrogel. Another aspect investigated in this study was the dissolving medium.

Traditionally, and for many years, sodium or potassium hydroxide have been used as alkaline solutions in the isolation of humic substances from solid-phase source materials, including isolation procedures used by International Humic Substances Society (De Nobili et al. 1990; Thurman and Malcolm 1981; Watanabe and Kuwatsuka 1991). Some researchers (Conte and Piccolo 1999; Gerzabek et al. 1997; Maia et al. 2008; Novotny et al. 1999; Nuzzo et al. 2013; Tatzber et al. 2007) used Na_4_P_2_O_7_ solution as an alternative extraction agent or in combination with the traditional NaOH solution. Tatzber et al. (2007) characterized in detail soil humic acids isolated using three different extractants including sodium hydroxide and tetrasodium pyrophosphate. Extraction using NaOH exhibited lower yields and probably extracted a different fraction of soil organic matter in comparison with the other extraction agents. They noted that sodium hydroxide can induce some reactions in organic matter like the Kolbe reaction, autooxidation, the breakdown of humic (macro)molecules, and the condensation of amino-carbonyls, as well as reactions like the hydrolysis of ester groups or the base-catalysed aldol condensation reaction (Head and Zhou 2000; Rosa et al. 2005; Tatzber et al. 2007).

In general, tetrasodium pyrophosphate is considered a milder agent for the extraction of humic acids. However, it might also lead to changes in the humic structure due to the incorporation of orthophosphate in humic fractions of lower molecular weights and pyrophosphate in humic fractions of higher molecular weights (Francioso et al. 1998; Tatzber et al. 2007). It is unknown whether differences in the analyses of humic acids extracted by different agents arose because of the extractions of different humic fractions or because of alterations to the extracted humic samples caused by the extractants themselves (Francioso et al. 1998; Head and Zhou 2000; Rosa et al. 2005; Tatzber et al. 2007). Although, Tatzber et al. (2007) concluded that NaOH extraction did not influence the analysis, we decided to use both NaOH and Na_4_P_2_O_7_, the solutions most frequently used for the extraction of humic substances, in our study. The dissolution of humic acids in these two different solutions and their precipitation/gelation using different agents such as acidic solutions and multivalent ions can provide several types of humic hydrogels. Incorporated ions (Ca, Mg, and Fe) as well as potential changes in the humic structure caused by different solvents should affect the mobility of copper(II) in hydrogels. We believe that investigation of the interactions of copper(II) ions and their diffusion simultaneously and directly in these hydrogels can provide knowledge useful for predicting the toxicity and mobility of trace metals in nature.

## Materials and methods

### Preparation of humic acids

Humic acids were isolated from South Moravian lignite (Czech Republic). They were prepared by means of an alkali extraction procedure using a mixture of 0.5 M NaOH and 0.1 M Na_4_P_2_O_7_ (1:1). This initial step was followed by centrifugation, the precipitation of humic acids from the alkali-soluble portion by a solution of HCl (pH < 2), washing, purification, and drying. The procedure is described elsewhere (Klučáková 2014; Klučáková and Kalina 2015; Peuravuori et al. 2006). More details on the chemical structure of the initial lignite matrix, as well as that of the isolated humic sample, can be found in previous works (Peuravuori et al. 2006, 2007). The basic characteristics of the humic sample are listed in Table 1.

**Table 1:** Basic characteristics of humic acids

C_*a*_ (at. %)	H_*a*_ (at. %)	O_*a*_ (at. %)	N_*a*_ (at. %)	S_*a*_ (at. %)	Total acidity (mmol g^−1^)	COOH (mmol g^−1^)
32.3	39.0	27.0	1.4	0.3	4.9	3.7

### Preparation of humic hydrogels

The powdered humic acids were dissolved in 0.5 M NaOH solution (hydrogels H) or 0.1 M Na_4_P_2_O_7_ solution (hydrogels P). The ratio between the solid humic sample and the solution was 8 g per 1 dm^3^. The solutions were used for the preparations of all hydrogels as described below.

Basic hydrogels (H and P) were prepared by the precipitation method—the hydrogels were created after collapse of the humic sols, when their ionic groups were neutralized, and electrostatic repulsion was suppressed. The solutions of humic acids in hydroxide and pyrophosphate were acidified by concentrated HCl solution up to a pH value close to 1. The precipitated humic hydrogels were washed repeatedly with deionized water and centrifuged until Cl^−^ ions were removed.

Hydrogels with incorporated metal ions were prepared by precipitation of the hydroxide and pyrophosphate solutions by 1 M CaCl_2_, 1 M MgCl_2_, and 1 M FeCl_3_. The volume ratio between humic solution and precipitation solution was 1:1. It is known that cations of weak hydroxides can hydrolyze in water, which causes acidic behavior in solutions of their salts. In the case of prepared hydrogels, we assume that the equilibrium of this reaction is shifted to the left as a result of the liberation of H^+^ ions from humic acids (Klučáková and Pekař 2009). The precipitations of hydroxides were not observed in the preparation of hydrogels (in contrast to blank experiments, in which NaOH solutions were added to salt solutions). The precipitated hydrogels were washed repeatedly by deionized water and centrifuged until Cl^−^ ions were removed. They were designated as H-Ca, H-Mg, and H-Fe (hydrogels based on NaOH solution); and as P-Ca, P-Mg, and P-Fe (hydrogels based on Na_4_P_2_O_7_ solution).

Hydrogels were characterized by means of FT-IR spectrometry (Nicolet iS5, Thermo Scientific). They were dried and the spectra of xerogel (solid matter) were collected using KBr pellets as described previously (Haberhauer and Gerzabek 1999; Tatzber et al. 2007).

### Diffusion from an instantaneous planar source

The hydrogels were pressed gently into glass tubes (length 3 cm and diameter 1 cm). A circular piece of filter paper (diameter 1 cm) was sunk into the 1 M CuCl_2_ solution for 1 min and then added to one side of the tube filled by hydrogel. The tube was wrapped with parafilm and aluminum foil to prevent the hydrogel drying. The durations of the diffusion experiments were 5, 24, 48, and 72 h, respectively. Then, the glass tube was removed, the hydrogel was sliced, and both the paper and hydrogel slices were extracted separately in 1 M HCl solution (Klučáková et al. 2014; Klučáková and Kalina 2015). The concentration of Cu^2+^ ions in leachate was determined by means of UV/VIS spectrometry (Hitachi U3900H). The obtained data were used to compute the concentration profiles of metal ions in the tubes and their diffusion fluxes (the total amounts diffused into the hydrogel). All experiments were performed at laboratory temperature (25 ± 1 °C) and in triplicate. Data are presented as average values with standard deviation bars.

### Diffusion from a constant source

The hydrogels were packed gently into glass tubes (length 5 cm and diameter 1 cm). The tubes were immersed in a saturated solution of CuCl_2_ (150 cm^3^)_._ Vessel lids were sealed with parafilm to prevent evaporation. The durations of the diffusion experiments were 5, 24, 48, and 72 h. Then, each tube was taken out from the solution, the glass tube was removed, the hydrogel was sliced, and both the paper and hydrogel slices were extracted separately in 1 M HCl solution (Klučáková and Pekař 2003b, 2004). The concentration of metal ions in leaches was determined by means of UV/VIS spectroscopy (Hitachi U3900H). The obtained data were used to compute the concentration profiles of metal ions in the tubes and their diffusion fluxes (the total amounts diffused into the hydrogel). All experiments were performed at laboratory temperature (25 ± 1 °C) and in triplicate. Data are presented as average values with standard deviation bars.

## Results and discussion

### Humic hydrogels

**Fig. 1:**
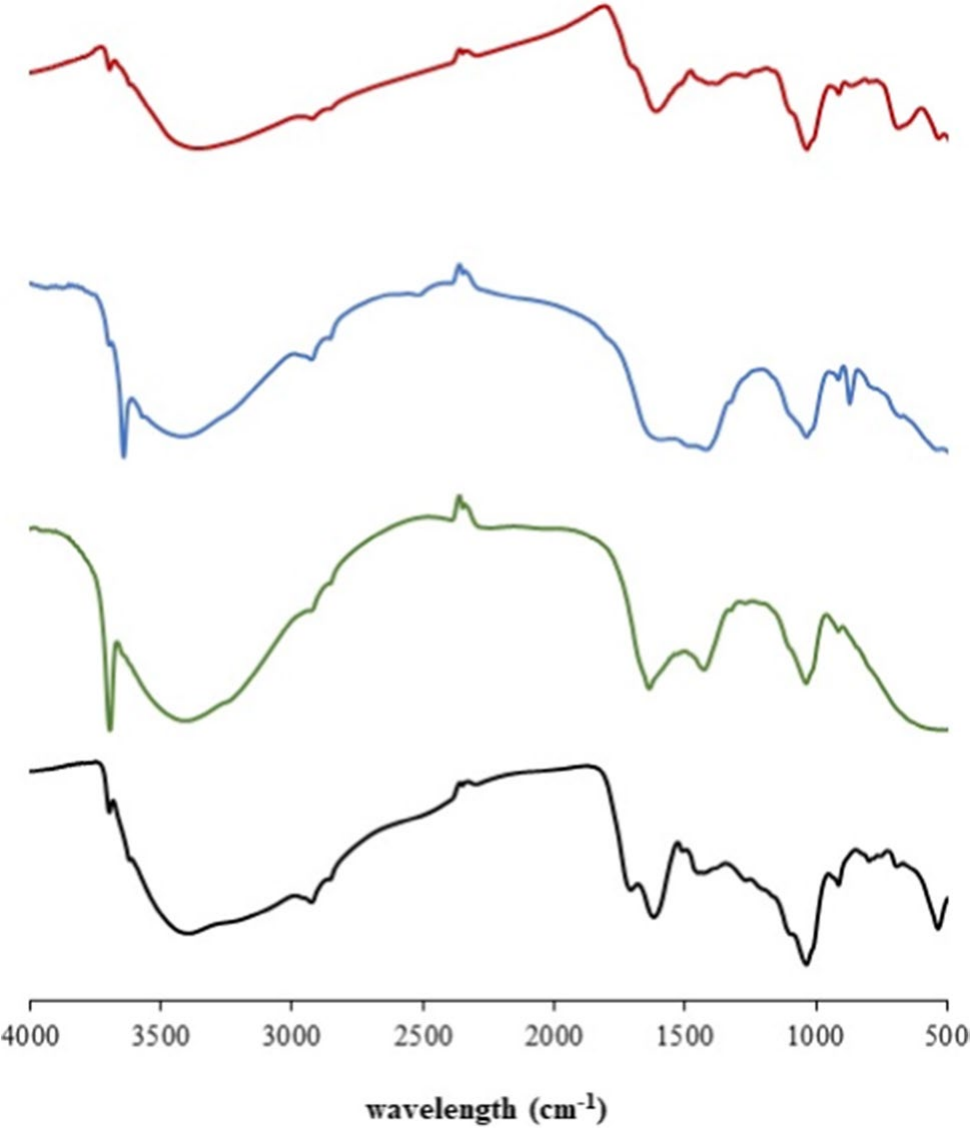
FT-IR spectra (transmittance) of xerogel samples based on sodium hydroxide: H (black), H-Ca (blue), H-Mg (green), and H-Fe (red)

**Fig. 2:**
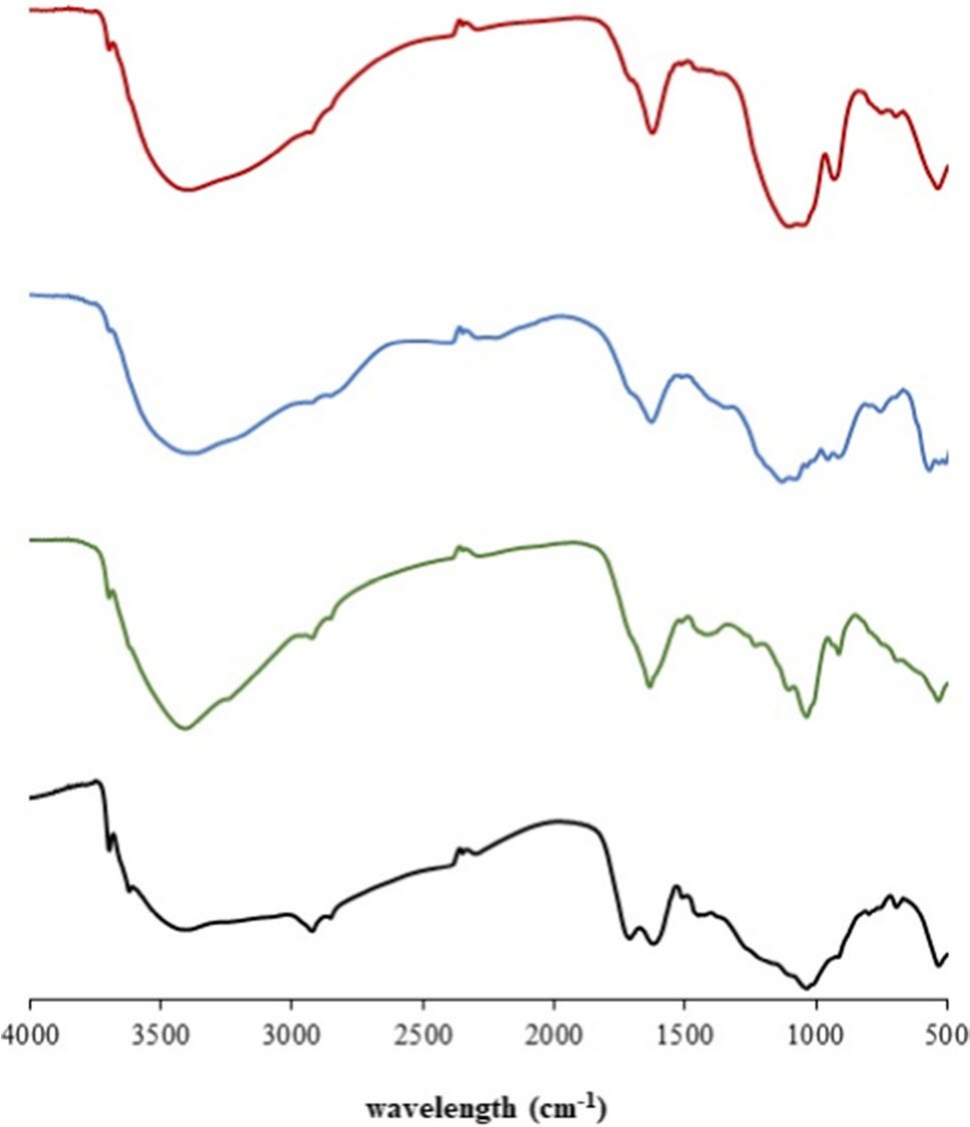
FT-IR spectra (transmittance) of xerogel samples based on tetrasodium pyrophosphate: P (black), P-Ca (blue), P-Mg (green), and P-Fe (red)

Prepared hydrogels were characterized by means of FT-IR spectrometry. The spectra shown in Figs. 1 and 2 show that the use of different solvents resulted in changes of spectra. In spite of the fact that humic acids were isolated from lignite by a mixture of NaOH and Na_4_P_2_O_7_, their re-dissolving in one of these solvents can influence the character of the gelled sample. As mentioned above, tetrasodium pyrophosphate is considered a milder agent for the extraction of humic acids, while sodium hydroxide is considered a stronger extractant which can induce some reactions in organic matter (Tatzber et al. 2007). According to previous studies (Francioso et al. 1998; Head and Zhou 2000; Rosa et al. 2005; Tatzber et al. 2007), differences in the analyses of humic acids extracted by different agents result from the extraction of different humic fractions or from alterations of the extracted humic samples by the extractants themselves. In our case, changes to the spectra should be caused only by an alteration of the humic sample. Changes were recorded mainly in the area < 1800 cm^−1^, which can be considered as the region exhibiting the predominant characteristics of humic acids, where almost all vibrational information on humic backbones can be identified (Chen et al. 2015). The bands around 1620 cm^−1^ (a C = O band with a possible contribution of nitrates and nitrites) and 1450 cm^−1^ (aliphatic C-H) were stronger in the case of xerogel based on hydroxide, and the spectrum showed a higher resolved fingerprint area. In this area, the OH deformation bands (1410–1260 cm^−1^), ester bands (1330 and 1050 cm^−1^), and fingerprint bands of aromatic structures (1225–950 cm^−1^) can be found. It should be noted that the area around 1000 cm^−1^ can be influenced by inorganic impurities in the samples. Similar differences were observed in spectra published by Tatzber et al. (2007). The incorporation of metal ions into humic samples influenced the spectra mainly in the same area (< 1800 cm^−1^). Stronger bands around 3600 cm^−1^ in the spectra of the H-Ca and H-Mg hydrogels can be assigned to free OH groups (although precipitations of hydroxides were not observed in the preparation of hydrogels). Changes in spectra differed both for individual metal ions and the solvent used for the preparation of hydrogel. In contrast, the common characteristic of xerogels with incorporated metal ions was the practical disappearance of the band of carbonyl vibration in carboxylic groups, aldehydes, ketones, and esters (~ 1700 cm^−1^) (or it was observed only as a shoulder). Two strong bands (around 1600 and 1400 cm^−1^), which were observed in the spectra of xerogels with incorporated metal ions, may be attributed to the asymmetric and symmetric stretches stretching of COO^−^ groups, respectively (Paradelo et al. 2012; Sowers et al. 2018). This can indicate that binding through COO^−^ groups may be a mechanism for the incorporation of metals into humic structure. Although some of the bands overlap with uncertain assignments, it can be seen that the incorporation of metal ions into the humic sample resulted in specific changes in the measured spectra and that the characteristic bands changed with the addition of different metals.

### Diffusion from an instantaneous planar source

**Fig. 3:**
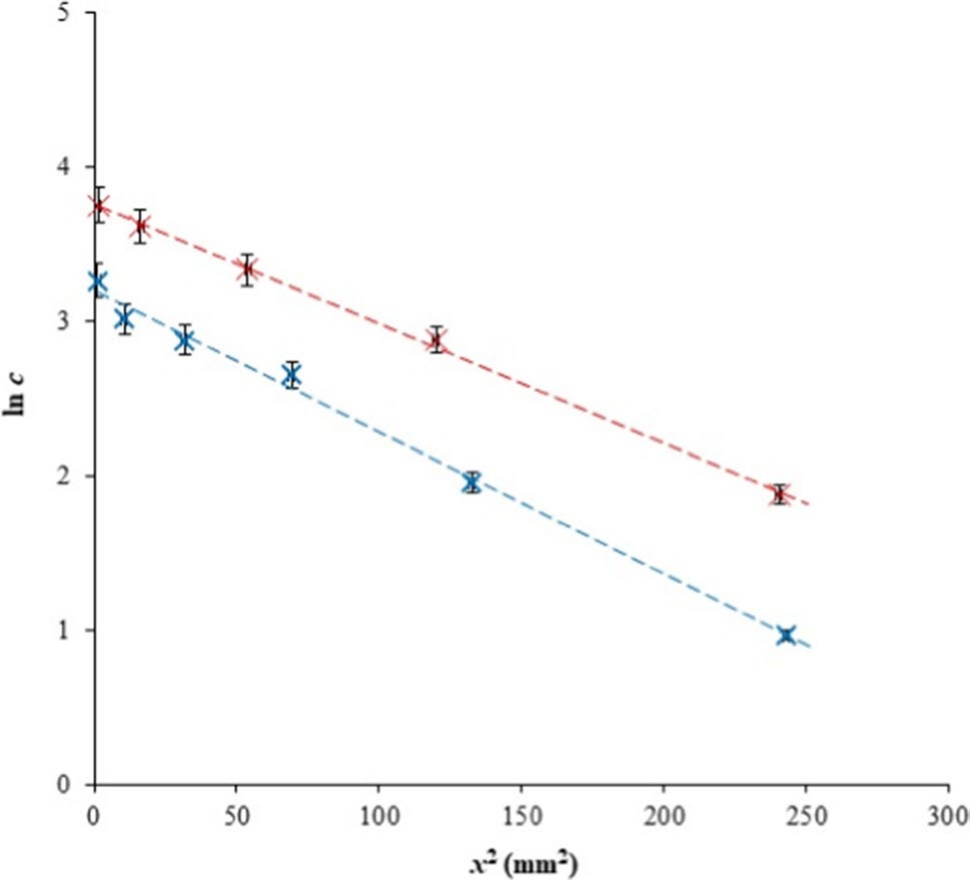
Example of data fitting for the method of instantaneous planar source—Eq. 2: H hydrogel after 24 h (blue) and P hydrogel after 24 h (red)

This method is based on the placement of a small, predefined amount of diffusing substance as a diffusion source on the surface of the diffusion medium. In this study, the source was the filter paper saturated by diffusing particles and applied on the frontal circular surface of the hydrogel in the tube. The particles then diffuse into the hydrogel and their surface concentration decreases (because the source is exhausted relatively soon). At equilibrium, the homogeneous distribution of metal ions in the hydrogel is achieved. This means that the concentration of metal ions is then constant and independent of the distance from the interface. The concentration profile in equilibrium is thus a line parallel with the x-axis. The concentration in the hydrogel is much lower in comparison with the method of constant source discussed below. Therefore, in this study, the potential dependence of diffusivity on the content of diffusing particles in the hydrogel could be investigated. The mathematical model applied to the data was based on the presumption that the diffusion was not close to equilibrium, and that the diffusion trajectories were shorter that the length of the hydrogel. Simultaneously, the amount of metal ions applied on the interface was known and no metal ions were present in the hydrogel at the start of the experiment. The final solution of second Fick’s second law is (Klučáková 2022; Klučáková et al. 2014; Klučáková and Kalina 2015):

1 $$c=\frac{n}{S\sqrt{\pi {D}_{ef}t}}exp\left(-\frac{{x}^{2}}{4{D}_{ef}t}\right),$$

where *c* is the concentration of diffusing particles, *n* is the total mass of the diffusing substance applied in the form of a narrow pulse, *S* is the cross-section available for diffusion,* D*_*ef*_ is the effective diffusion coefficient, *t* is time, and *x* is the distance from the interface between the filter paper and the hydrogel. The equation is usually applied in its logarithmic form:

2 $$\text{ln}c=\text{ln}\frac{n}{S\sqrt{\pi {D}_{ef}t}}-\frac{{x}^{2}}{4{D}_{ef}t},$$

which can be used for the calculation of the effective diffusion coefficient* D*_*ef*_. In Fig. 3, the example of experimental data fitted by Eq. 2 is shown. The effective diffusion coefficients were calculated from the slopes of the lines. The obtained values are listed in Tables 2 and 3. They are average values for all diffusion times (durations of experiments) in three replications and include both the influence of the pore hydrogel structure and the chemical interactions of copper with the hydrogel. The pore structure can be characterized by the porosity *φ* and the tortuosity *τ* (Klučáková 2022; Klučáková et al. 2014; Klučáková and Kalina 2015). The porosity is the proportion of the pore volume in the total volume of hydrogel. If we assume that particles can diffuse only through pores in the hydrogel and that diffusion into the (solid) hydrogel network is negligible, the area of hydrogel accessible for them is smaller in comparison with a homogeneous medium.

**Table 2:** Effective diffusion coefficients determined by Eq. 2 for hydrogels based on hydroxide

*D*_*ef, H*_ (m^2^ s^−1^)	*D*_*ef, H-Ca*_ (m^2^ s^−1^)	*D*_*ef, H-Mg*_ (m^2^ s^−1^)	*D*_*ef, H-Fe*_ (m^2^ s^−1^)
(3.78 ± 0.18) × 10^−10^	(1.62 ± 0.12) × 10^−10^	(1.51 ± 0.13) × 10^−10^	(2.74 ± 0.15) × 10^−10^

**Table 3:** Effective diffusion coefficients determined by Eq. 2 for hydrogels based on pyrophosphate

*D*_*ef, P*_ (m^2^ s^−1^)	*D*_*ef, P-Ca*_ (m^2^ s^−1^)	*D*_*ef, P-Mg*_ (m^2^ s^−1^)	*D*_*ef, P-Fe*_ (m^2^ s^−1^)
(3.45 ± 0.13) × 10^−10^	(1.30 ± 0.10) × 10^−10^	(1.18 ± 0.09) × 10^−10^	(2.60 ± 0.16) × 10^−10^

A further effect is that the diffusion rate is dependent on the shape and real length of the diffusion pathway. Because the pores are not straight, the diffusion takes place more effectively over a longer distance than it would in a homogenous material. The tortuosity is a value characterizing the longer distance traversed in the pores. The structural parameter µ (= *φ / τ*) represents the influence of the structure of humic hydrogel and its local geometry in the diffusion (Klučáková 2022; Klučáková et al. 2014; Klučáková and Kalina 2015). If the hydrogel contains an active substance able to interact with diffusing particles, transport through the hydrogel is affected by these interactions and a proportion of the particles can be immobilized by the chemical reaction. The effective diffusion coefficient thus includes the effect of such interactions (besides the influence of the pore structure of the hydrogel). Interactions between humic acids and metal ions are relatively complex. Humic acids can be described as a mixture of many different organic substances containing different types of binding sites. A detailed description of the interactions is not possible. On the other hand, we can assume that the interactions are much faster in comparison with the diffusion. Therefore, a local equilibrium between immobilized and free movable Cu(II) ions can be attained. This means that the front of diffusing ions is in contact with the hydrogel containing non-occupied binding sites and (simultaneously) that this passing front is equilibrated from the point of view of the chemical reaction between humic acids and diffusing Cu(II) ions. If this simple equilibrium between immobilized and free movable Cu(II) ions is assumed, an apparent equilibrium constant *K* can be defined as the ratio between the concentrations of these two forms:

3 $$K=\frac{{c}_{im}}{{c}_{free}},$$

and included into the value of the effective diffusion coefficient (together with structural factor µ):

4 $${D}_{ef}=D\frac{\varphi }{\tau \left(K+1\right)}=D\frac{\mu }{K+1},$$

where *D* is the diffusion coefficient of Cu(II) ions in water.

The values of the effective diffusion coefficient obtained by means of the method of instantaneous planar source are listed in Tables 2 and 3. If we compare values obtained for hydrogels based on hydroxide (Table 2) and pyrophosphate (Table 3), we can see that the effective diffusion coefficients are lower for pyrophosphate cross-linked by different agents (HCl, CaCl_2_, MgCl_2_, FeCl_3_). However, the differences are in some cases comparable with the measuring error (e.g., H-Fe and P-Fe). The highest values of *D*_*ef*_ were obtained for pure hydrogels (without incorporated metals), the lowest ones for hydrogels containing incorporated magnesium. The effective diffusion coefficients obtained for hydrogels without incorporated metal ions are much higher than values obtained for hydrogels enriched by metals but lower than the diffusion coefficient of Cu(II) ions in water (Haynes 2012). This means that both the real length of the diffusion pathway in hydrogel pores and the interactions of Cu(II) ions with humic acids resulted in a decrease in diffusion coefficients.

Differences between *D*_*ef*_ values obtained for hydrogels based on hydroxide and pyrophosphate can be connected with structural changes to the humic acids. Francioso et al. (1998) reported that pyrophosphate could be incorporated in the structure of humic acids in their extraction process. Similarly, Tatzber et al. (2007) observed differences in the structural characteristics of humic acids extracted by pyrophosphate and other reagents. In contrast to the mentioned studies (Francioso et al. 1998; Tatzber et al. 2007), the humic acids used in this work were extracted by a mixture of NaOH and Na_4_P_2_O_7_ solutions. Then, the final powdered humic sample was used for the preparation of hydrogels. Differences in the properties of hydrogels based on hydroxide and pyrophosphate thus resulted from re-dissolving humic acids in one of the reagent solutions during hydrogel preparation.

As described above, differences in the FT-IR spectra of xerogels based on hydroxide and pyrophosphate were observed. The differences were similar to those published for the extraction of humic acids by means of various reagents (Francioso et al. 1998; Tatzber et al. 2007). This means that the structure and properties of humic acids can be changed by their re-dissolving. It is probable that the structural differences are connected with differences in the diffusion characteristics of hydrogels. The potential incorporation of pyrophosphate into the humic structure can result in changes in the permeability of the hydrogel as well as changes in its affinity to copper(II) ions. Both effects can cause a decrease in diffusion coefficients, and it is not easy to determine which effect predominates. If metal ions such as Ca(II), Mg(II), and Fe(II) were incorporated into hydrogels during their preparation, the decrease in the diffusion coefficient was much more significant. As described above, the presence of the mentioned metal ions can affect the binding of copper to organic matter (e.g., Gao et al. 2015; Hamilton-Taylor et al. 2002; Kogut and Voelker 2001; Muller and Cuscov 2017; Saito et al. 2005). Many authors have observed a decrease in the binding of copper in the presence of calcium and magnesium (Gao et al. 2015; Kang et al. 2010; Kogut and Voelker 2001). Wu and Hendershot (2010) confirmed the positive effect of calcium with respect to the suppression of copper toxicity.

In contrast, Hamilton-Taylor et al. (2002) showed that the presence of calcium and magnesium had no measurable effect. Our results show that the presence of calcium and magnesium suppressed the mobility of copper(II) ions in humic hydrogels. This indicates that calcium and magnesium can promote reactivity of humic hydrogels. It is known that the competition between copper and magnesium (and calcium) can decrease copper binding as a result of there being lower amounts of binding sites available for copper(II) ions (Gao et al. 2015; Kang et al. 2010). The effects observed in our experiments were different. The diffusion rate decreased as a result of chemical interactions and changes in the hydrogel structure. The most effective metal in our study was magnesium, closely followed by calcium. We assume that the metals are incorporated in the humic structure and occupy a proportion of humic binding sites. The stability of humic complexes with magnesium and calcium is lower in comparison with copper (Dinu 2015; Evangelou and Marsi 2001; Wieckowska and Martyniuk 2003). Therefore, metal ions can be replaced by copper(II) ions in the diffusion through humic hydrogel. Rey-Castro et al. (2009) derived the effective distribution of affinities of metal ions to humic substances under natural water conditions. They stated that calcium and magnesium display a greater effective affinity for carboxylic functional groups while the distributions of trivalent iron and copper between carboxylic and phenolic groups are overlapped. They computed that the affinity of Ca and Mg to carboxylic binding sites was higher than the affinity of Cu and Fe(III). These findings show that the presence of calcium and magnesium can suppress the interactions of copper with humic substances. In contrast, our results indicate that the diffusivity of Cu(II) ions significantly decreases if calcium and magnesium are incorporated into humic hydrogel. This means that their presence can suppress the permeability of the hydrogel and support its interactions with copper. The influence of the incorporation of Fe(II) ions was weaker but still significant. It can be connected with the different affinity to humic functional groups described above. In order to confirm or disprove the potential support of interactions between humic hydrogel and Cu(II) ions in the presence of other metals, the method of diffusion from a constant source was used for subsequent experiments.

### Diffusion from a constant source

This method is based on a constant concentration at the interface between the hydrogel and the donor solution. In contrast to the previous method (diffusion from a coinstantaneous planar source), a higher amount of Cu(II) ions diffused into the hydrogel. Therefore, the potential dependence of the diffusion coefficient on concentration could be investigated. In this study, the source was a super-saturated solution, i.e., it was a saturated solution with a small amount of undissolved salt on the bottom of vessel. This salt was able to be dissolved when copper(II) ions diffused into the hydrogel, thus resulting in the constant concentration of the donor solution. The mathematical model applied to the data was based on the presumption of a semi-infinite medium. This means that the diffusion trajectories were shorter that the hydrogel dimension. The final solution of Fick’s second law is (Klučáková 2022; Klučáková et al. 2014; Klučáková and Kalina 2015):

5 $$c={c}_{s} erfc\frac{x}{2\sqrt{{D}_{ef}t}},$$

where *c* is the concentration of diffusing particles, *c*_*s*_ is the concentration at the interface between the hydrogel and the donor solution, *S* is the cross section available for diffusion,* D*_*ef*_ is the effective diffusion coefficient, *t* is time, and *x* is the distance from the interface. The total amount of Cu(II) ions diffused into the hydrogel (*m*_*t*_) can be expressed as (Klučáková 2022; Klučáková et al. 2014; Klučáková and Kalina 2015):

6 $${m}_{t}=2{c}_{s}\sqrt{\frac{{D}_{ef}t}{\pi }}$$

**Fig. 4:**
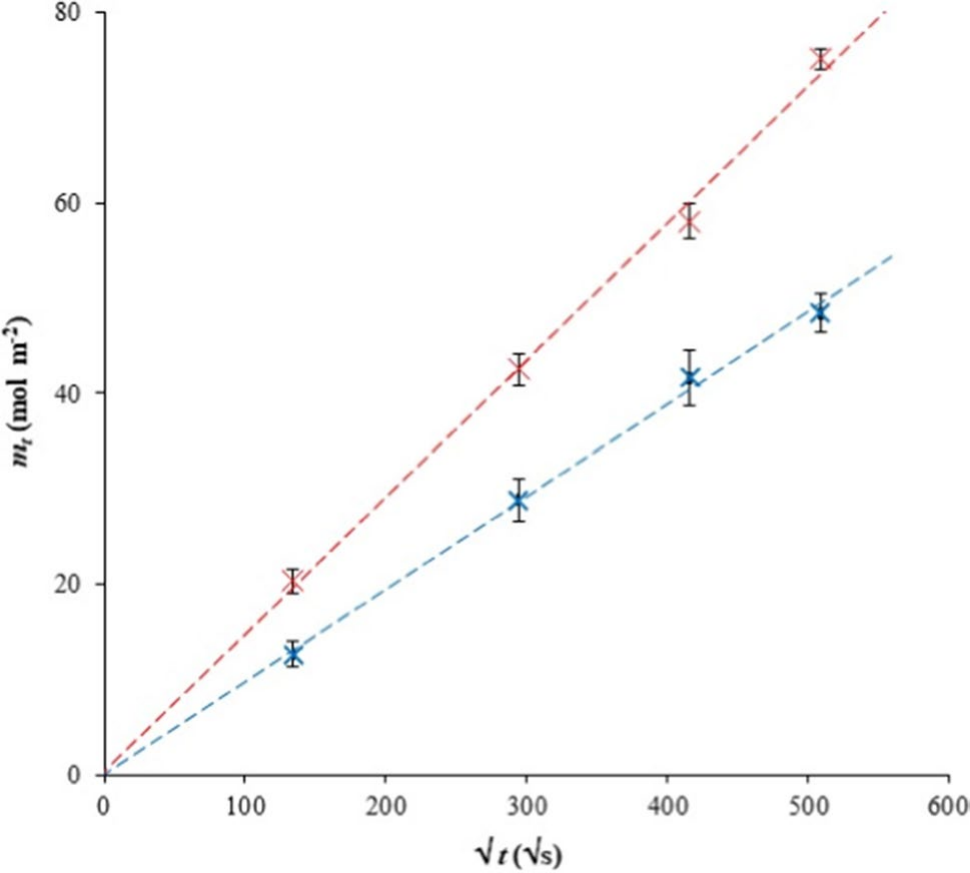
Example of data fitting for the method of constant source—Eq. 6: H hydrogel (red) and H-Ca hydrogel (blue)

The effective diffusion coefficient* D*_*ef*_ can be calculated from the slope of the linear dependence of *m*_*t*_ against the square root of time. In Fig. 4, the experimental example fitted by Eq. 6 is shown. The effective diffusion coefficients obtained by the method of constant source are listed in Tables 4 and 5. As can be seen, they are higher in comparison with the method of instantaneous planar source (Tables 2 and 3). This means that the diffusion coefficient is dependent on the concentration of diffusing particles. The diffusion rate is affected not only by the diffusion coefficient and also by the gradient of concentration. It means that lower diffusion coefficient and very high concentration gradient can result in higher diffusion rate than system with very low concentration gradient and high diffusion coefficient. Both parameters are higher in the case of the constant diffusion source; therefore, the diffusion rate should also be higher.

**Table 4:** Effective diffusion coefficients determined by Eq. 6 for hydrogels based on hydroxide

*D*_*ef, H*_ (m^2^ s^−1^)	*D*_*ef, H-Ca*_ (m^2^ s^−1^)	*D*_*ef, H-Mg*_ (m^2^ s^−1^)	*D*_*ef, H-Fe*_ (m^2^ s^−1^)
(9.91 ± 0.37) × 10^−10^	(6.89 ± 0.36) × 10^−10^	(5.63 ± 0.29) × 10^−10^	(1.68 ± 0.04) × 10^−9^

**Table 5:** Effective diffusion coefficients determined by Eq. 6 for hydrogels based on pyrophosphate

*D*_*ef, P*_ (m^2^ s^−1^)	*D*_*ef, P-Ca*_ (m^2^ s^−1^)	*D*_*ef, P-Mg*_ (m^2^ s^−1^)	*D*_*ef, P-Fe*_ (m^2^ s^−1^)
(9.61 ± 0.05) × 10^−10^	(6.53 ± 0.22) × 10^−10^	(6.17 ± 0.28) × 10^−10^	(1.67 ± 0.26) × 10^−9^

**Fig. 5:**
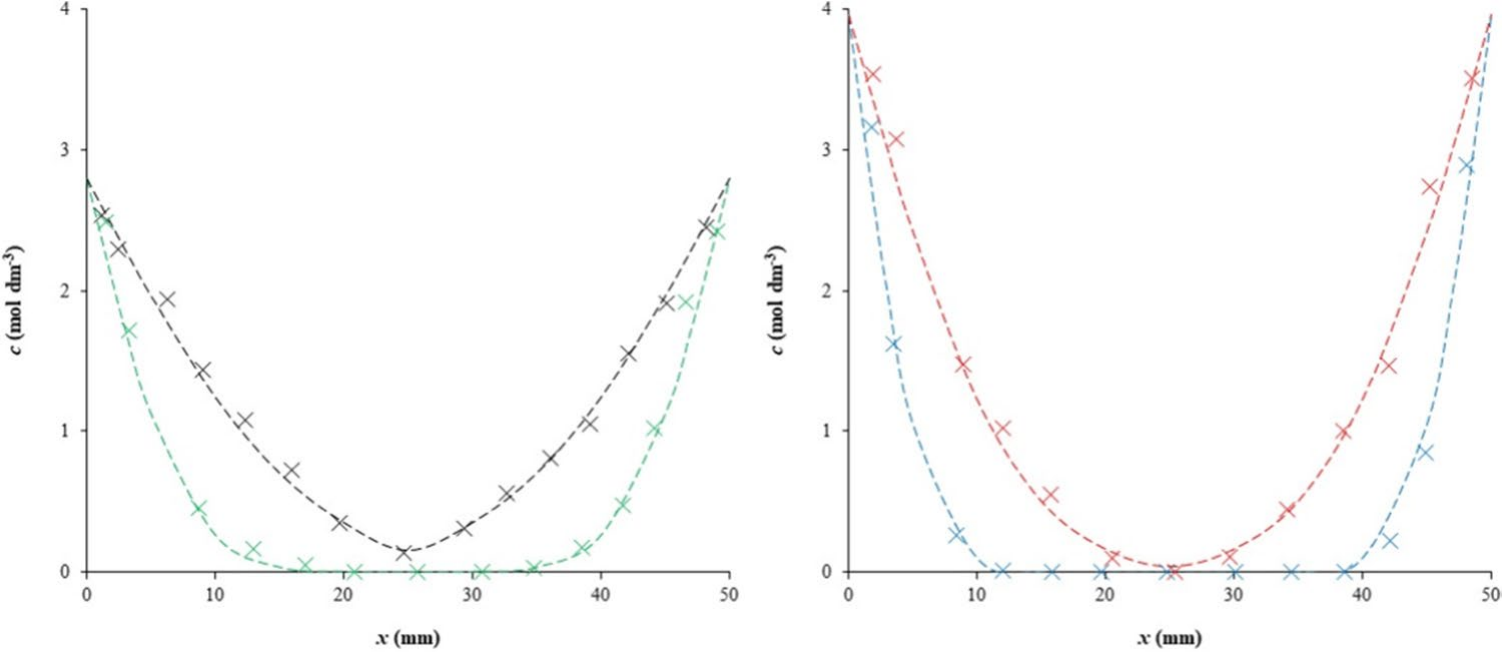
Examples of concentration profiles in H (left) and H-Ca (right) hydrogels for the method of constant source. Data are fitted by Eq. 5: H hydrogel after 5 h (green) and 24 h (black), H-Ca hydrogel after 5 h (blue) and 24 h (red)

As described above, the diffusion is strongly affected by the pore structure of the hydrogel and the interactions of diffusing particles with active substance incorporated in the hydrogel. In our study, the pore structure in the hydrogel was the same for both diffusion methods used and was determined by the type of hydrogel. In contrast, interactions between diffusing particles and active sites in the hydrogel can be affected by the concentration of reactants as well as the ionic strength. It is known that the rate of the chemical reaction is determined by the rate constant and the concentration of reactants (Monk 2004). An increase in concentration results in a higher reaction rate. The simple model of the local equilibrium between immobilized and free movable Cu(II) ions (Eq. 3 and Eq. 4) assumes that interactions are much faster than diffusion and that the system is (at a given place) equilibrated immediately. On the other hand, the equilibrium should be considered to be dynamic, because new diffusing particles are continuously passing through the given place. Therefore, the concentration of Cu(II) ions at a given place increases in time, as can be seen in Fig. 5, and local equilibrium is continually reestablished. It is known that the presence of other ions can affect reaction rates in aqueous medium. This phenomenon, called the primary salt effect, can cause both an increase and decrease in the reaction rate. The final effect depends on the charge of the reacting ions (Monk 2004). If they have the same charge, the reaction should be promoted and its rate increased.

The effect of iron on the reaction kinetics in humic systems was also observed by other authors (Ding et al. 2019; Farina et al. 2018; Lippold et al. 2007; Shi et al. 2016). On the other hand, the primary salt effect can affect only the reaction rate. This means that the equilibrium should remain unaltered, but the system should be equilibrated in a shorter time.

However, the salt effect can influence the diffusivity of copper in hydrogel. The mechanisms of interactions between metal ions and humic substances are much more complex than a simple equilibrium between free movable and immobilized metal ions. There are many different binding sites in the structure of humic substances which differ in their chemical character and bond strength. The simplification which is represented by the assumption of the rapid achievement of local equilibrium can be considered as a useful tool for characterizing the transport properties of metal ions in humic systems. It cannot describe the complex process of diffusion connected with interactions in detail but can provide information about the mobility and amount of free movable metal ions. Figure 5 shows another effect of the incorporation of metal ions into hydrogel. We can see that the surface concentration of copper(II) ions differs for individual types of hydrogels. This confirms that the structure of hydrogels changed with the incorporation of metal ions into them. The concentration ratio at the phase interface between hydrogel and donor solution can thus be considered as resulting from the hydrogel structure and the ability of copper to interact with the hydrogel.

## Conclusions

The migration behavior of metal ions in natural systems and their interactions with organic matter are crucial for determining their toxicity and bioavailability. We developed methods of mobility and reactivity mapping (Klučáková 2014; Klučáková et al. 2014; Klučáková and Kalina 2015; Klučáková and Pekař 2003a, b, 2004, 2009) which allow investigation of the transport of diffusing particles and their interactions with active substances directly with respect to their motion through the studied system. This study took into account the real situation in soils, which subsumes the existence of many organic and inorganic constituents. While previous studies were focused on the role of organic matter and humic substances in the migration and bioavailability of metal ions, the novelty of this study is the reactivity and transport mapping in systems containing complexed ions naturally occurring in soils (such as Ca, Mg, and Fe). The presence of Ca, Mg, and Fe in organo-mineral complexes can affect the biotoxicity of heavy metals (such as copper), whose accumulation in soil poses risks to human and ecosystem health (Christl et al. 2005; Wu and Hendershot 2010); for example, increased contents of calcium can suppress the uptake of copper by pea roots (Wu and Hendershot 2010). Our results confirm that the incorporation of Ca, Mg, and Fe into humic systems can strongly affect the mobility of copper in humic hydrogels. While the incorporation of Ca and Mg resulted in the decrease in the diffusivity of copper ions, the incorporation of Fe(III) into humic hydrogel resulted in an increase in the diffusivity of Cu(II) in the hydrogel in comparison with pure humic hydrogel. Diffusion coefficients based on the method of diffusion from constant source were higher in comparison with the instantaneous planar source which confirmed their concentration dependence. The use of pyrophosphate in the preparation of humic hydrogels resulted in moderate decrease in diffusivity of copper.

The effective diffusion coefficients determined by our methods include both the influence of the structure of the diffusion medium and the effects of interactions between diffusing particles and active substances in the medium. Our findings suggest that the soil type and its composition can affect the mobility of toxic pollutants and their uptake by plants. The current study has major implications for future research into the bioavailability of toxic metals in soils. It provides basic characteristics of the diffusivity of copper in humic hydrogels containing natural metals such as calcium, magnesium, and iron. Further investigations are required to determine how the mobility of copper (and other toxic metals) can be affected by the different contents of natural ions and organic matter.

## Data Availability

Data will be made available on request.
